# Prognostic factors for improvement of shoulder function after arthroscopic rotator cuff repair: a systematic review

**DOI:** 10.1016/j.jseint.2022.09.003

**Published:** 2022-09-29

**Authors:** Thomas Stojanov, Laurent Audigé, Linda Modler, Soheila Aghlmandi, Christian Appenzeller-Herzog, Rafael Loucas, Marios Loucas, Andreas Marc Müller

**Affiliations:** a Department of Orthopaedic Surgery and Traumatology, University Hospital of Basel, Basel, Switzerland; b Research and Development, Shoulder and Elbow Surgery, Schulthess Clinic, Zurich, Switzerland; c Basel Institute for Clinical Epidemiology and Biostatistics, University Hospital Basel and University of Basel, Basel, Switzerland; d University Medical Library Basel, University of Basel, Basel, Switzerland; e Department of Orthopedics, Balgrist University Hospital, University of Zurich, Zurich, Switzerland

**Keywords:** Prognostic factors, Risk factors, Arthroscopy, Rotator cuff tear, Shoulder function, Systematic review, Epidemiology

## Abstract

**Background:**

The identification of factors that specify prognostic models for postoperative results should be based on the best scientific evidence and expert assessment. We aimed to identify, map, and evaluate potential prognostic factors for the improvement of shoulder function in patients undergoing arthroscopic rotator cuff repair.

**Methods:**

Longitudinal primary studies of arthroscopic rotator cuff repair reporting any multivariable factor analyses for shoulder function improvement with an endpoint assessment of at least 6 months were included. We systematically searched EMBASE, Medline, and Scopus for articles published between January 2014 and June 2021. The risk of bias of included studies and the quality of evidence were assessed using the Quality in Prognosis Studies tool and an adapted Grading of Recommendations, Assessment, Development, and Evaluations framework.

**Results:**

Overall, 24 studies including 73 outcome analyses were included. We classified younger age and smaller tear size as probably prognostic for a greater improvement in objective outcomes. Shorter symptom duration, absence of a worker compensation claim, low preoperative level of functional status, and high preoperative pain level were classified as probably prognostic for greater improvement in patient-reported outcome measures. The quality of the synthesized evidence was low. Twenty-one studies had an overall high risk of bias.

**Conclusion:**

Six potential prognostic factors for shoulder function after arthroscopic rotator cuff repair were identified. Along with ongoing expert opinion assessments, they will feed into a prognostic model-building process.

## Rationale

In the field of arthroscopic rotator cuff repair (ARCR), clinicians base their recommendation for surgery on multiple factors including the patient's potential for shoulder function improvement. A Swiss multicenter ARCR cohort was implemented to develop and validate clinical prediction models for key postoperative outcomes including shoulder function improvement,^2^ which ultimately support an evidence-based decision-making process. However, the development and validation of such clinical prediction models require a cautious research strategy,^29^ that begins with the identification of factors specifying the clinical prediction models and relies on both expert assessment and literature review.^59^ Attempts were already made to identify potential prognostic factors for shoulder function improvement after ARCR, yet the interpretation was limited by low quality underlying evidence.^21^^,^^34^^,^^39^^,^^44^^,^^47^^,^^57^^,^^63^ To complement these initial efforts and to account for the increasing number of recently published articles in the field, a state-of-the-art systematic review of the latest literature was needed. Thus, we aimed to comprehensively identify, map, and evaluate potential prognostic factors for the improvement of shoulder function in patients undergoing ARCR.

## Methods

The present review was written according to the updated Preferred Reporting Items for Systematic Review and Meta-Analysis guidelines.^52^ The protocol was registered in PROSPERO on August 24, 2020 (registration number: CRD42020199257). Detailed methods were described elsewhere.^64^

Briefly, longitudinal primary studies of adult patients who underwent primary ARCR that reported on multivariable factor analyses for shoulder function improvement with an endpoint assessment of at least 6 months were included. Shoulder function outcomes were classified as objective outcomes (including muscle strength and range of motion parameters), or patient-reported outcome measures (PROMs) (including all the patient-reported shoulder function scales, such as the American Shoulder and Elbow Surgeons (ASES)^45^ scale, the Constant-Murley^11^ score, the Simple Shoulder Test^41^ (SST), University of California Los Angeles^1^ shoulder score, the Western Ontario Rotator Cuff (WORC)^31^ score and its short version (short WORC),^16^ the Oxford Shoulder Score,^13^ the Japanese Orthopedic Association or the visual analog scale (VAS) for shoulder pain).

A systematic search was run in EMBASE (Elsevier), Medline (Ovid), and Scopus for articles published between 2014 and June 9, 2021 (see Supplemental File 1). Search results were limited to 2014 and onward, since surgical rotator cuff repairs substantially evolved in 2013/2014.^14^ To complement the results of database searching, we implemented a screening of all the included references as well as the citing articles of those indexed in Scopus or the Web of Science (June 10, 2021). The bibliographic references of identified topical systematic reviews and research articles were also screened as an additional source.

Two screening phases based on titles and abstracts and full-texts, respectively, were performed independently by two authors (TS, LM) and involved the judgment of a senior author (LA), when necessary. Data extraction and risk of bias assessment using the Quality in Prognosis Study tool^28^ were performed independently by pairs of two authors (TS, LM, ML, and RL). Data extraction items were based on an adaptation of the Checklist for Critical Appraisal and data extraction for systematic reviews of prediction modeling studies for prognostic factors (see Supplemental File 2).^46^

Effect estimates were reported as described in individual studies. The quality of the synthesized evidence was graded according to an adaptation of the Grading of Recommendations, Assessment, Development, and Evaluations framework applied to prognostic factors findings.^33^ Potential prognostic factors were then narratively synthesized in the Results section when the quality of the evidence was “Low.” We raised the quality assessment of the synthesized evidence when 50% or more of the studies reported the same direction for an association between a given factor and its outcome.

Based on this quality assessment, factors were then categorized into patient-related, disease-related, and procedure-related factors with potential prognostic value or as requiring further analyses.

## Results

**Figure 1:**
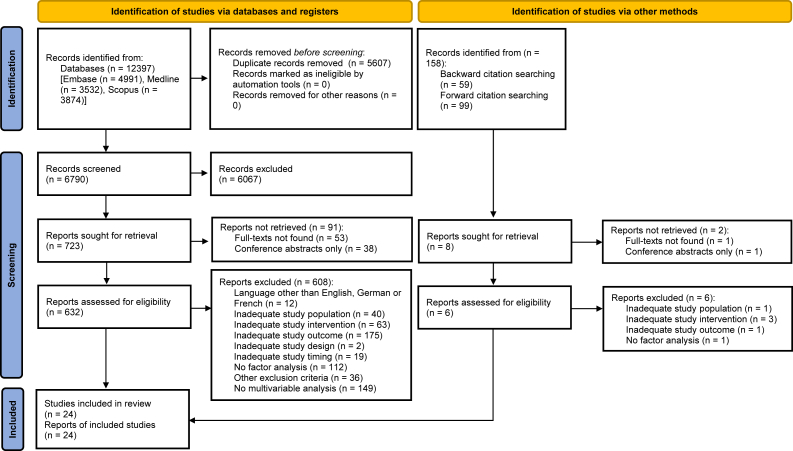
PRISMA 2020 flow diagram for new systematic reviews which included search of databases, register, and other sources. *PRISMA*, Preferred Reporting Items for Systematic Review and Meta-Analysis.

We screened the titles and abstracts of 6790 records and assessed 632 full-text articles for eligibility (Fig. 1). We finally included 24 studies^3^^,^^4^^,^^6^^,^^12^^,^^17^, ^18^, ^19^, ^20^^,^^22^^,^^25^^,^^27^^,^^36^^,^^37^^,^^43^^,^^48^^,^^49^^,^^51^^,^^54^^,^^56^^,^^61^^,^^65^^,^^66^^,^^68^^,^^69^ representing 5830 patients. We excluded two recent studies including patients with revision repairs or nonoperative treatment.^5^^,^^24^ Screening of the titles and abstracts of cited and citing references of included records and 18 topical records^7^, ^8^, ^9^^,^^15^^,^^23^^,^^24^^,^^26^^,^^30^^,^^35^^,^^40^^,^^42^^,^^55^^,^^58^^,^^60^^,^^70^, ^71^, ^72^, ^73^ did not yield any additional studies that met our inclusion criteria. A full description of included studies (studied population, outcomes, statistical analyses, and reported effect estimates) is available in Supplemental Tables 1-3.

### Study characteristics

Among the included studies, 11 (46%) were conducted in the United States of America^4^^,^^6^^,^^12^^,^^17^, ^18^, ^19^^,^^25^^,^^48^^,^^56^^,^^68^^,^^69^ and one (4%) in Europe.^22^ Patient sample sizes ranged from 30^22^ to 1600^61^ (Table I). Only one-fourth were prospective studies.^22^^,^^37^^,^^48^^,^^56^^,^^65^^,^^69^

**Table I:** Synthesized study characteristics.

Included studies (N = 24)	No. (%)	Associated references
Sample size		
1-100	13 (54)	^6^^,^^17^^,^^18^^,^^22^^,^^27^^,^^36^^,^^37^^,^^48^^,^^49^^,^^51^^,^^56^^,^^65^^,^^69^
101-300	8 (33)	^3^^,^^4^^,^^12^^,^^19^^,^^25^^,^^43^^,^^54^^,^^68^
301-1000	1 (4)	^20^
1001+	2 (8)	^61^^,^^66^
Prospective study (vs. retrospective)	6 (25)	^22^^,^^37^^,^^48^^,^^56^^,^^65^^,^^69^
Types of tears included		
All	17 (71)	^3^^,^^4^^,^^6^^,^^12^^,^^22^^,^^27^^,^^37^^,^^43^^,^^48^^,^^49^^,^^51^^,^^54^^,^^56^^,^^61^^,^^65^^,^^68^
Supraspinatus and/or infraspinatus only	4 (17)	^17^^,^^20^^,^^36^^,^^69^
Supraspinatus only	3 (12)	^18^^,^^19^^,^^66^
Etiology		
All	22 (92)	^3^^,^^4^^,^^6^^,^^12^^,^^17^, ^18^, ^19^, ^20^^,^^22^^,^^27^^,^^36^^,^^37^^,^^43^^,^^49^^,^^51^^,^^54^^,^^56^^,^^61^^,^^65^^,^^66^^,^^68^^,^^69^
Traumatic only	1 (4)	^25^
Degenerative only	1 (4)	^48^
Partial/full-thickness tears		
All	14 (58)	^6^^,^^12^^,^^17^^,^^22^^,^^27^^,^^36^^,^^37^^,^^43^^,^^49^^,^^51^^,^^54^^,^^61^^,^^65^^,^^66^^,^^69^
Full-thickness only	8 (34)	^3^^,^^4^^,^^20^^,^^25^^,^^48^^,^^56^^,^^68^^,^^69^
Partial-thickness only	2 (8)	^18^^,^^19^
Number of surgeons involved		
One surgeon	9 (37.5)	^3^^,^^6^^,^^19^^,^^20^^,^^22^^,^^37^^,^^61^^,^^65^^,^^66^
Two surgeons	3 (12.5)	^17^^,^^51^
Three surgeons or more	8 (33)	^12^^,^^25^^,^^43^^,^^48^^,^^54^^,^^56^^,^^68^^,^^69^
Missing information	4 (17)	^4^^,^^18^^,^^36^^,^^49^
Outcome type		
Continuous	16 (67)	^6^^,^^18^, ^19^, ^20^^,^^22^^,^^25^^,^^27^^,^^43^^,^^48^^,^^54^^,^^56^^,^^61^^,^^65^^,^^66^^,^^69^
Dichotomous∗	7 (29)	^12^^,^^17^^,^^36^^,^^37^^,^^49^^,^^51^^,^^68^
Categorized	1 (4)	^66^
End point		
6 mo	2 (8)	^61^^,^^66^
12 mo	13 (54)	^4^^,^^6^^,^^12^^,^^18^^,^^19^^,^^22^^,^^36^^,^^37^^,^^43^^,^^54^^,^^56^^,^^68^^,^^69^
24 mo	9 (37)	^3^^,^^17^^,^^20^^,^^25^^,^^27^^,^^48^^,^^49^^,^^51^^,^^65^

∗ Authors used thresholds to dichotomize their outcomes with the achievement of minimal clinical important differences (MCID), patient acceptable symptom state (PASS), substantial clinical benefit (SCB), or maximal outcome improvement (MOI).

Of the 24 included studies, 17 (71%) included all types of rotator cuff tears (supraspinatus, infraspinatus, and/or subscapularis tears),^3^^,^^4^^,^^6^^,^^12^^,^^22^^,^^25^^,^^27^^,^^37^^,^^43^^,^^48^^,^^49^^,^^51^^,^^54^^,^^56^^,^^61^^,^^65^^,^^68^ and 22 (92%) reported outcomes for both degenerative and traumatic tears.^3^^,^^4^^,^^6^^,^^12^^,^^17^, ^18^, ^19^, ^20^^,^^22^^,^^25^^,^^27^^,^^36^^,^^37^^,^^43^^,^^49^^,^^51^^,^^54^^,^^56^^,^^61^^,^^65^^,^^66^^,^^68^^,^^69^ Fourteen studies (58%) reported outcomes for all types of tears (including full or partial-thickness tears).^6^^,^^12^^,^^17^^,^^22^^,^^27^^,^^36^^,^^37^^,^^43^^,^^49^^,^^51^^,^^54^^,^^61^^,^^65^^,^^66^^,^^69^ The number of surgeons involved in individual studies ranged from one^3^^,^^6^^,^^19^^,^^20^^,^^22^^,^^37^^,^^61^^,^^65^^,^^66^ to six.^12^

Continuous outcomes were reported in 16 studies (67%),^6^^,^^18^, ^19^, ^20^^,^^22^^,^^25^^,^^27^^,^^43^^,^^48^^,^^54^^,^^56^^,^^61^^,^^65^^,^^66^^,^^69^ whereas dichotomous outcomes reported in 7 studies (29%),^12^^,^^17^^,^^36^^,^^37^^,^^49^^,^^51^^,^^68^ respectively. Postoperative outcome time point assessments were made at 6 months for two studies^61^^,^^66^ (8%), 12 months for 13 studies^4^^,^^6^^,^^12^^,^^18^^,^^19^^,^^22^^,^^36^^,^^37^^,^^43^^,^^54^^,^^56^^,^^68^^,^^69^ (54%), and 24 months for nine studies^3^^,^^17^^,^^20^^,^^25^^,^^27^^,^^48^^,^^49^^,^^51^^,^^65^ (37%).

Due to heterogeneity in reported outcomes and prognostic factor definitions, we were not able to perform meta-analysis as originally planned during review registration.

### Objective outcomes

Five studies reported objective outcomes^20^^,^^61^^,^^65^^,^^66^ (Table II), including postoperative abduction strength,^61^^,^^66^ external rotation strength,^61^^,^^66^ internal rotation,^66^ adduction strength^66^ at 6 months and 24 months.^20^ Range of motion in external rotation at 6^61^^,^^66^ and 24 months,^65^ forward flexion at 6^65^ and 24 months,^65^ abduction at 6 months,^66^ and internal rotation at 6 months were also reported.^66^

**Table II:** Synthesized study outcomes.

Unique analyses∗ (n = 73)	No. (%)	Associated references
Objective outcomes	14 (19)	^31^^,^^43^, ^44^, ^45^
Muscle strength	7 (9.5)	^31^^,^^43^^,^^45^
Range of motion	7 (9.5)	43, 44, 45
Patient-reported outcome measures	59 (81)	24, 25, 26, 27, 28, 29, 30, 31, 32, 33, 34, 35, 36, 37, 38, 39, 40, 41, 42^,^^45^, ^46^, ^47^
American Shoulder and Elbow Surgeons score	16 (22)	25, 26, 27^,^^29^, ^30^, ^31^^,^^33^^,^^36^^,^^37^^,^^42^^,^^46^^,^^47^
Shoulder pain	15 (20)	^24^^,^^26^^,^^29^^,^^30^^,^^33^^,^^35^^,^^36^^,^^42^^,^^45^, ^46^, ^47^
Constant Score	7 (10)	^24^^,^^27^^,^^29^, ^30^, ^31^
Subjective Shoulder Value	6 (8)	^27^^,^^33^^,^^36^
Simple Shoulder Test	4 (7)	^26^^,^^33^^,^^42^^,^^46^
University of California Los Angeles score	3 (4)	^37^^,^^40^^,^^41^
Western Ontario Rotator Cuff score	3 (4)	^28^^,^^38^^,^^47^
Oxford Shoulder Score	2 (3)	^24^^,^^34^
Perceived-shoulder Hindrance	1 (1)	^32^
Japanese Orthopedic Association shoulder score	1 (1)	^39^
Short- Western Ontario Rotator Cuff score	1 (1)	^31^

Each outcome was studied separately, and results were reported for each analysis.

∗ One single article might report different factor analyses for different outcomes.

### Prognostic factors for objective outcomes

Overall, 23 potential prognostic factors for objective outcomes were identified and included 12 patient-related factors, 7 disease-related factors, and 4 procedure-related factors (Table III and see Supplemental Table 3a).

**Table III:** Summary of prognostic factor findings for objective outcomes.

Factor category	Probably prognostic (low quality of evidence)	Requiring further analyses (very low quality of evidence)
Patient-related	Increasing age^20^^,^^61^	Difficulty with behind the back activity^61^ Difficulty with overhead activity^61^ Hypertension^65^ Lymphocyte monocyte ratio^65^ Preoperative muscle strength^20^^,^^61^ Preoperative overall shoulder satisfaction^61^ Preoperative pain level^61^ Preoperative range of motion^61^ Preoperative perceived stiffness^61^ Sex^61^^,^^65^ Worker’s compensation claim^20^
Disease-related	Larger tear size^20^^,^^61^	Concomitant rotator cuff pathologies^20^ Tear severity^61^ Tear size^20^^,^^61^ Tendon mobility^61^ Tissue quality^61^ Traumatic onset^66^
Procedure-related		Number of anchors^61^ Operative time^61^ Repair quality^61^ Surgical technique^65^

### Younger age

Two studies reported significant associations between age and postoperative objective outcomes. The first study reported a multivariable test result for dichotomized age categories of smaller than 55 years old or greater than 55 years old, which indicated that increasing age was significantly associated with worse postoperative objective outcome (*P* < .0001).^20^ The second study reported a regression coefficient (β) of −0.227 (*P* = .008) ^61^ for increasing age that was kept as a continuous factor (Table III and see Supplemental Table 3). Both results suggested that younger age was associated with greater improvement in postoperative objective outcomes.

### Smaller tear size

Results from two studies suggested that smaller tear size was associated with greater improvement in postoperative objective outcomes; when described as the largest tear dimension measured intraoperatively and categorized as small (less than 1 cm), medium (1 to 3 cm), and large (3 to 5 cm), authors reported a significant multivariable association (*P* < .0001)^20^ and, when kept continuous and expressed as area (in cm2), authors reported a regression coefficient of β = −0.332 for increasing tear size (*P* = .006).^61^

### PROMs

A total of 22 studies reported on postoperative or changes in PROMs^3^^,^^4^^,^^6^^,^^12^^,^^17^, ^18^, ^19^, ^20^^,^^22^^,^^25^^,^^27^^,^^36^^,^^37^^,^^43^^,^^48^^,^^49^^,^^51^^,^^54^^,^^56^^,^^66^^,^^68^^,^^69^ (Table II).

### Prognostic factors for PROMs

Overall, 48 potential prognostic factors were identified including 12 patient-related factors, 18 disease-related factors, and 18 procedure-related factors (Table IV and see Supplemental Table 3b).

**Table IV:** Summary of prognostic factor findings for patient-reported outcome measures.

Factor category	Probably prognostic (low quality of evidence)	Requiring further analyses (very low quality of evidence)
Patient-related	Shorter symptom duration^29^^,^^30^^,^^33^^,^^35^^,^^40^ Worker’s compensation claim^25^^,^^27^^,^^31^^,^^33^^,^^40^^,^^46^	Age^26^, ^27^, ^28^, ^29^, ^30^, ^31^^,^^33^^,^^36^, ^37^, ^38^^,^^40^, ^41^, ^42^^,^^46^^,^^47^ Alcohol use^28^ ASA classification^26^^,^^42^ Body mass index^26^^,^^27^^,^^32^^,^^33^^,^^42^^,^^47^ Depression and anxiety^42^ Diabetes^27^^,^^28^^,^^37^^,^^40^^,^^47^ Sex^27^^,^^30^^,^^33^^,^^36^, ^37^, ^38^^,^^41^^,^^42^^,^^46^^,^^47^ Smoking status^26^, ^27^, ^28^^,^^36^^,^^37^^,^^40^^,^^42^^,^^47^ Hypertension^27^^,^^37^^,^^40^ Temperament^24^
Disease-related	Higher preoperative functional scores^27^^,^^31^^,^^33^^,^^34^^,^^36^^,^^37^^,^^40^ Higher preoperative pain level^35^^,^^36^^,^^39^^,^^40^	Acromion type^41^ Dominance affected side^25^^,^^27^^,^^37^^,^^40^ Concomitant rotator cuff pathologies^31^ Cuff tear index^38^ Fatty infiltration^26^^,^^32^^,^^38^^,^^40^ Preoperative muscle strength^40^ Preoperative range of motion^32^^,^^39^ Postoperative shoulder stiffness^35^ Postoperative retear^36^ Synovitis^35^ Tear location^29^^,^^30^ Tear pattern^25^^,^^27^^,^^35^^,^^36^ Tear retraction^26^^,^^32^^,^^37^^,^^40^^,^^42^ Tear shape^47^ Tear size^26^, ^27^, ^28^, ^29^^,^^31^^,^^33^, ^34^, ^35^, ^36^^,^^40^, ^41^, ^42^^,^^46^^,^^47^ Traumatic onset^29^^,^^30^^,^^40^^,^^45^
Procedure-related		Acromioclavicular joint procedures^27^^,^^41^ Acromioplasty^35^, ^36^, ^37^^,^^41^ Biceps procedure^25^, ^26^, ^27^^,^^36^^,^^37^^,^^40^^,^^41^ Concomitant procedures^30^ Follow-up duration^40^ Infraspinatus repair^37^ Lateral debridement^27^ Mobilization^27^ Number of anchors^26^^,^^28^ Preoperative corticosteroid injections^29^ Preoperative physical therapy^29^^,^^30^ Posterosuperior tear repair^41^ Procedure location^26^ Repair technique^26^^,^^27^^,^^36^ Subscapularis repair^26^^,^^37^ Supraspinatus repair^37^ Surgeon effect^28^ Timing of preoperative corticosteroid injection^30^

### Shorter symptom duration

Five studies reported associations between symptom duration and postoperative PROMs.^18^^,^^19^^,^^25^^,^^36^^,^^51^ Six multivariable outcome-factor analyses (50%) reported a significant association. One study reported a 19-point better improvement in Constant Score at 12 months (β = 19.4; *P* < .001) in patients undergoing the operation within 3 months after symptom onset compared to other patients.^19^ In another study, performing the operation within 3 months after symptom onset was associated with a 3-times higher odds (odds ratio = 3.1; 95% confidence interval 1.1 to 8.6; *P* = .028) to achieve a patient acceptable symptom state corresponding to a value of 1.7 points in VAS shoulder pain.^36^ In the third study, three outcome analyses were reported, a repair within 4 months after symptom onset resulted in 10.3 points improvement in 24 months ASES (*P* = .008), 1.8 points in 24 months SST (*P* = .001), 8.6 points improvement in Subjective Shoulder Value (*P* = .033), and 0.93 points improvement in pain VAS scale (*P* = .028).^25^ One study reported a trend of less improvement in shoulder function after longer symptom duration without reaching statistical significance on multivariable analysis.^18^ Altogether, these results suggested that shorter symptom duration was associated with greater improvement in PROMs.

### Absence of a worker’s compensation claim

Six studies reported associations between the worker’s compensation claim status and PROMs.^4^^,^^12^^,^^20^^,^^25^^,^^51^^,^^68^ Of the 20 outcome-factor analyses reported, 9 (45%) multivariable associations were reported.^4^^,^^12^^,^^20^^,^^25^ One study reported an association between the presence of a worker’s compensation claim and worse postoperative Constant Score, short WORC, and ASES at 24 months (*P* < .0001).^20^ Two studies reported significant odds ratio suggesting the presence of a worker’s compensation claim was associated with worse improvement in PROM.^4^^,^^12^ One study reported a 11-point lower ASES at 12 months in patients with a worker’s compensation claim (β = −11.1; *P* = .019).^25^ Three multivariable associations were, however, not statistically significant (*P* = .061 for postoperative 24 months SST score, *P* = .071 for postoperative 24 months Subjective Shoulder Value score, and *P* = .055 for postoperative 24 months VAS pain score).^25^ These results suggested that the presence of a worker’s compensation claim was associated with lower improvement in PROMs.

### Worse preoperative functional status

Associations between baseline levels of functional status or pain level were studied in 36 analyses across nine studies,^12^^,^^20^^,^^25^^,^^27^^,^^36^^,^^37^^,^^43^^,^^49^^,^^51^ 17 analyses reported significantly lower shoulder function improvement in patients with higher preoperative functional status^12^^,^^25^^,^^27^^,^^37^^,^^43^ and 4 analyses reported significant associations between higher preoperative pain level and better postoperative PROMs.^36^^,^^37^^,^^49^ The synthesized results indicated that worse preoperative functional status (including higher baseline pain levels) was associated with greater improvement in PROMs.

### Quality of the synthesized evidence

The overall quality of the evidence was low to very low. Nonetheless, younger age and smaller tear size were classified as probably prognostic for greater improvement in objective functional outcomes, yet with a low quality of evidence (Table III). Shorter symptom duration, absence of a worker compensation claim, and worse baseline functional status (including higher baseline pain levels) were classified as probably prognostic for greater improvement in PROMs (Table IV). The quality of the synthesized evidence on prognostic factor findings was notably affected by the absence of a full reporting of prognostic factor estimates.

### Risk of bias

**Figure 2:**
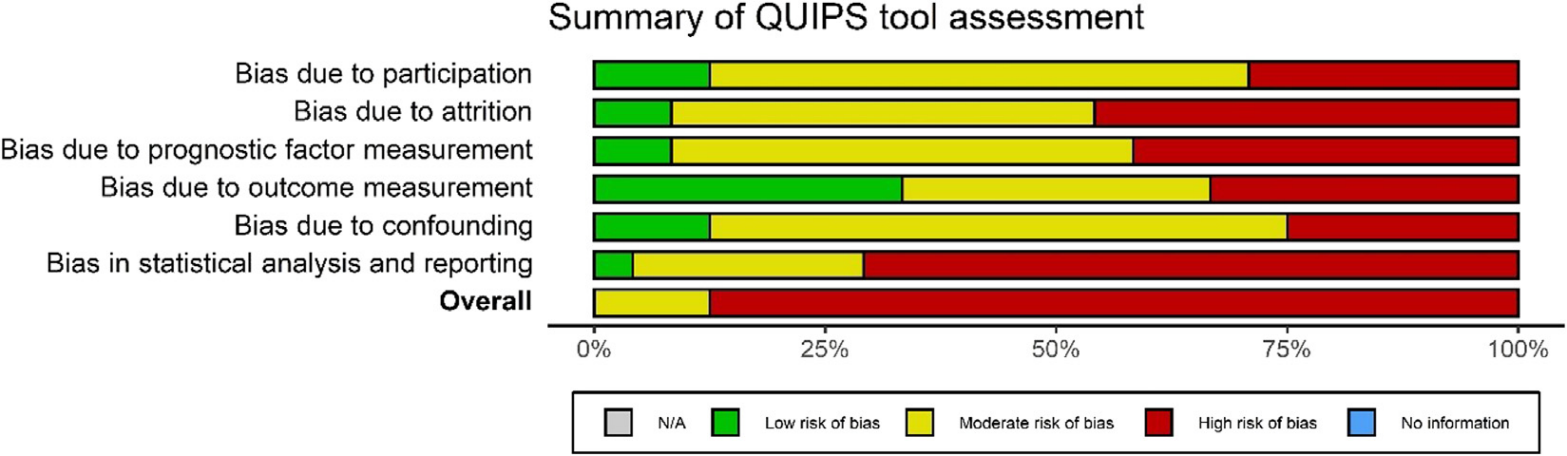
Summary of QUIPS tool assessment. *QUIPS*, Quality in Prognosis Study.

Three studies (12.5%) had an overall moderate risk of bias^25^^,^^37^^,^^69^ with the remaining studies judged as having an overall high risk of bias (Fig. 2, see Supplemental Table 4). This assessment was notably impacted by the item “Statistical Analysis and Reporting,” mostly due to a lack of appropriate multivariable and univariable effect estimates reporting.

### Multivariable modeling phase

Only studies with a low or moderate risk of bias in the item “Statistical Analysis and Reporting” were considered in this section, representing 7 studies (29.1%) and 32 outcome analyses^4^^,^^6^^,^^12^^,^^25^^,^^37^^,^^48^^,^^69^ (Table V and see Supplemental Table 5). Some working groups included all the initial factors presented in their analyses in the reported multivariable models,^25^^,^^48^^,^^69^ whereas others included factors in their reported multivariable models on the basis of significant univariable^12^^,^^36^ or multivariable analyses^6^ (29% and 14%, respectively). Lastly, one study (4%) reported a performance indicator for their presented model based on the Hosmer-Lemeshow goodness of fit test.^37^

**Table V:** Synthesized study modeling phase.

Independent articles with low or moderate risk of bias for the item “statistical analysis and reporting” (N = 7)	N (%)	Associated references
Criteria to include factors in presented multivariable model		
Significant on univariable analysis	2 (29)	^12^^,^^36^
Significant on multivariable analysis	1 (14)	^6^
Stepwise regression	1 (14)	^4^
All factors were included	3 (43)	^25^^,^^48^^,^^69^
Presented model performance indicators		
None	6 (86)	^4^^,^^6^^,^^12^^,^^25^^,^^48^^,^^69^
Hosmer-Lemeshow test	1 (14)	^37^

## Discussion

The objective of the present review was to identify, map, and evaluate potential prognostic factors for the improvement of shoulder function in patients undergoing ARCR. We classified younger age and a smaller tear size as probable prognostic factors for greater improvement in objective outcomes. The absence of a worker compensation claim, shorter symptom duration, and worse baseline functional status (including higher preoperative levels of pain) was classified as probable prognostic factors for greater improvement in PROM.

### General interpretation of the results in the context of other evidence

During the preparation of our manuscript, a confirmatory systematic review and meta-analysis with slightly different inclusion criteria was published, reporting that prospective ARCR studies with lower mean outcome values at baseline and smaller tear sizes were associated with better clinical outcomes.^32^ Other systematic review authors reported the existence of a correlation between poor baseline psychological function and worsening postoperative PROM^53^ and identified a wide variety of prognostic factors for functional clinical outcomes, but also conflicting evidence and low methodological quality of included studies.^21^^,^^39^^,^^44^^,^^57^^,^^63^ Still, Fermont et al concluded that younger age and smaller tear size was associated with better recovery,^21^ but could not classify the duration of symptoms as a prognostic factor. Lambers Heerspink et al identified increased age and larger tear size as negative predictors of functional status recovery, and the presence of a worker’s compensation claim as having a negative influence on functional outcomes. Again, however, duration of symptoms could not be classified as being prognostic due to limited evidence.^39^ Yet, duration of symptoms is a known predictor for worse baseline outcome status, indicating the confounding nature of this factor for baseline status.^38^ Such a factor should therefore be considered when describing baseline associations. McElvany et al reported that older patients and larger tears have an increased risk of failure of rotator cuff repair.^44^ Raman et al also reported a negative effect of larger tear size, increasing age, and worker’s compensation claim status on ARCR outcomes,^57^ but, again, the authors did not find a significant influence of symptom duration. Saccomanno et al reported that retear risk is affected by older age and larger tear size and that baseline scores and work compensation claims were the most significant predictors for functional outcomes.^63^ Taken together, our findings on prognostic factors are supported by similar previous reviews, with the notable exception of symptom duration, which was not identified by other systematic reviewers, probably due its confounding nature and to the heterogeneity in the sets of factors used to model postoperative outcomes.

### Modeling changes in functional outcomes

We defined an improvement in outcomes as an improvement in outcomes at a patient level, regardless of whether the reviewed studies focused on the achievement of minimal clinical important difference, the achievement of a patient acceptable symptom state or substantial clinical benefit, or whether postoperative values were modeled. Both indicators were relevant in our context because we aimed to identify blocks of factors influencing the change over time or postoperative values. However, we are aware of the impact that ceiling effects and preoperative functional status impact the achievement of minimal clinical important difference.^50^ When considering interpretable outcomes taking into consideration preoperative patient functional status, the use of a new indicator such as the maximal outcome improvement might be of importance, as defined by Beck et al.^4^ The benefits of the use of maximal outcome improvement are that a satisfactory outcome can be determined even for patients with high preoperative function and the challenges of ceiling effects restricted, especially when predicting interpretable outcomes for individual patients.^67^

### Limitations of the review processes used

Our review was limited by our choice to only analyze original articles published in English, German, and French. The risk of bias regarding the statistical analysis and reporting item was notably affected by the selective reporting of the included studies and focus on reporting only point effect estimates for significant associations. We would have expected the transparency of all univariable and multivariable regression coefficients to ensure a better understanding of the underlying associations between factors and outcomes. When published studies only report significant associations (at a *P* < .05 threshold), meaningful information regarding notable factors of estimated direction and strength of associations is missed. Having access to detailed and informative results might have permitted a meta-analysis on a given outcome for a given time point, yet this appeared inappropriate in the context of our review.

### Implications of the results for practice, policy, and future research

To improve current standards in the field, recommendations and a general framework for prognostic studies have been made.^29^ To improve the quality of reporting multivariable prognostic models, we foster the use of well-designed guidelines from the EQUATOR network group, such as the transparent reporting of a multivariable prediction model for individual prognosis or diagnosis statement.^10^

The results of our review are transferrable to the clinical setting and support the optimal decision-making process for surgery for a given patient. When aiming to achieve greater improvement after elective orthopedic surgery, a poor baseline patient status is usually a good indicator of success for improvement over time. However, this association is only observed for improvement in PROMs. In contrast, objective functional outcome measurements seem to decrease with greater tear size and older age. The same factors were shown to be associated with decreased tendon healing,^44^ which was found to be a relevant factor for the functional outcome, particularly for strength recovery.^62^ In clinical practice, patients with larger tear sizes and older age may therefore expect subjective recovery if their baseline PROMs are low, but they should be informed about limited functional improvements following ARCR and a high risk of retears. Namely, these patients may only be good candidates for ARCR if they have poor PROMs (particularly due to pain) with acceptable shoulder function. In contrast, surgery should not be delayed for young patients with small tear sizes given the high chances of functional improvement and potential negative effects of prolonged symptom duration.

## Conclusion

Six potential prognostic factors for shoulder function improvement were identified. Their prognostic value should be confirmed by expert assessment. The results of the present review are the initial step toward developing prediction models in ARCR outcomes as part of our ARCR_Pred cohort study.^2^

## Disclaimers:

Funding: This literature search is supported by the 10.13039/501100001711Swiss National Science Foundation, Switzerland Grant ID 320030_184959/1.

Conflicts of interest: The authors, their immediate families, and any research foundation with which they are affiliated have not received any financial payments or other benefits from any commercial entity related to the subject of this article.
