# Supplementary material for: Prognostic factors for improvement of shoulder function after arthroscopic rotator cuff repair: a systematic review
Source: JSES Int. 2022 Sep 29;7(1):50–7. doi: 10.1016/j.jseint.2022.09.003 (PMC9937854; doi:10.1016/j.jseint.2022.09.003)
Supplement: Supplemental Table 1 [file mmc3.docx]

**Supplemental Table 1: Detailed study characteristics and patient demographics**

| **Author** | **Year** | **Country** | **Design** | **Sample size** | **Participants** | **Intervention** | **Rehabilitation protocol** |
| --- | --- | --- | --- | --- | --- | --- | --- |
| Kim et al. ^36^ | 2014 | Korea (South) | R | 84 | Patients with traumatic and degenerative supraspinatus and infraspinatus tears;  Exclusion of patients with concomitant biceps lesions or acromioclavicular joint lesion, degenerative arthritis and inflammatory arthritis, or an inconsistent change of pain pattern | Intervention not precisely described | Not a standardized rehabilitation protocol, depending on the size of the tear |
| Dwyer et al. ^20^ | 2015 | Canada | R | 344 | Patients with full-thickness degenerative and traumatic supraspinatus or infraspinatus tears only with a symptom duration of at least 6 months;  Exclusion criteria included open or revision procedures, partial tears, and subscapularis tears. | One surgeon involved.  Single-row or double-row repair techniques used depending on the shape and the size of the tear | Post-operative immobilization during 6 weeks in a sling with passive and limited active range of motion (ROM) allowed. Active ROM and strengthening exercises started at 6 weeks after surgery. Full activity was allowed from 6 months |
| Fermont et al. ^22^ | 2015 | Netherlands | P | 30 | Patients with all types of partial or full-thickness rotator cuff tears, etiology not precisely describe;  Exclusion of patients requiring additional procedures (such as adhesive capsulitis, labrum lesion, osteoarthritis of the glenohumeral joint) or owing to Gouttalier stage superior or equal 3 | One surgeon involved.  Double-row, single-row or other repair techniques depending on the type of the tear. Other procedures (such as acromioplasty, distal clavicular resection, biceps tenotomy, or biceps tenodesis) were conducted | Post-operative immobilization during 6 weeks in an antirotation sling. Passive ROM exercises started the first day after the surgery under supervision of a physiotherapist. Strengthening of the shoulder started after 6 weeks |
| Pecora et al. ^54^ | 2015 | Brazil | R | 131 | Patients with all types of partial or full-thickness tears, etiology not precisely described;  Exclusion of patients with additional pathologic processes (such as glenohumeral instability, glenohumeral osteoarthritis, adhesive capsulitis, inflammatory arthropathy) | Five surgeons involved.  Repair technique not precisely described | Post-operative immobilization during 6 weeks in a sling. Passive ROM started after 3 weeks. Assisted active ROM started after 6 weeks. Strengthening exercises started after 3 months |
| Potter et al. ^56^ | 2015 | United States of America | P | 70 | Patients with full-thickness tears and etiology not precisely described;  No specific exclusion criteria | Three surgeons involved.  Single-row or double-row repair techniques used. Additional procedures (such as subacromial decompression, biceps tenotomy or tenodesis, glenohumeral debridement, and/or distal clavicle resection) were performed | Not precisely described |
| Donohue et al. ^18^ | 2016 | United States of America | R | 60 | Patients with high-grade partial-thickness supraspinatus tears, etiology not precisely described;  Exclusion of tears that extended into other rotator cuff tendons, and glenohumeral instability or adhesive capsulitis | Number of surgeons involved and repair technique not precisely described.  Additional procedures like distal clavicle excision and biceps tenotomy or tenodesis were done if necessary | Post-operative immobilization not described. Passive ROM started after 6 weeks. Assisted active ROM started after 6-8 weeks. Strengthening exercises started after 2 months. |
| Tan et al. ^66^ | 2016 | Australia | R | 1300 | Patients with supraspinatus tears, and both degenerative or traumatic tears;  No specific exclusion criteria | One surgeon involved.  Single-row repair technique used | Post-operative immobilization in a sling with a small abduction pillow. Active ROM started at 6 weeks after surgery. Strengthening exercises started after 3 months, and sport activities from 6 months |
| Donohue et al. ^19^ | 2017 | United States of America | R | 132 | Patients with high-grade supraspinatus partial-thickness tears;  Exclusion of tears extended into other rotator cuff tendons and patients with an experienced trauma to the affected shoulder within one year after surgery | One surgeon involved.  Repair technique not precisely described. Additional procedures (such as subacromial decompression, acromioclavicular resection, superior labrum anterior-posterior debridement, and biceps tenotomy or tenodesis) were conducted | Passive ROM was performed for the first 6 weeks, followed by active ROM from 6 to 8 weeks after surgery. Strengthening was then initiated at 8 weeks following surgery. |
| Ohzono et al. ^51^ | 2017 | Japan | R | 55 | Patients with large or massive rotator cuff tears and with intact tendons after surgery confirmed with magnetic resonance imaging;  Exclusion of patients with advanced glenohumeral arthritis, fractures of the shoulders or loss to follow-up | Two surgeons involved.  Single-row, double-row, or suture bridge technique depending on tendon mobility and tear configuration. Additional procedures (such as capsular release, tenotomy/tenodesis of the long head of the biceps tendon and distal clavicle excision) were conducted when needed | Post-operative immobilization in a sling with abduction pillow after surgery, with the shoulder internally rotated at 30 degrees to 40 and abducted at 20. Passive ROM exercise of the shoulder was commenced at postoperative day 4, and active ROM exercise was allowed at postoperative week 6. Isotonic muscle strengthening exercises were allowed at postoperative week 12. |
| Robinson et al. ^61^ | 2017 | Australia | R | 1600 | Patients with all types of rotator cuff tears, etiology not precisely described;  Exclusion of patients with concomitant procedures beside additive acromioplasty were excluded and patients with concurrent stabilization, capsular release or distal clavicle excision | One single surgeon involved.  Knotless inverted mattress repair technique with concurrent acromioplasty, depending on the shape and the size of the tear | Post-operative immobilization during 6 weeks in an abduction pillow. Passive ROM exercises started the eight day after the surgery. Strengthening of the shoulder started after 6 weeks. |
| Chalmers et al. ^6^ | 2018 | United States of America | R | 85 | Patients with all types of full-thickness tears;  No specific exclusion criteria | One single surgeon involved.  Single-row or trans osseous equivalent double-row repair | Post-operative immobilization during 6 weeks in a sling. Passive ROM started after 6 weeks. Assisted active ROM started after 6 weeks. Strengthening exercises started after 3 months. |
| Dierckman et al. ^17^ | 2018 | United States of America | R | 52 | Patients with degenerative or traumatic supraspinatus and infraspinatus tears, tear size between 2 and 4 cm and symptom duration of at least 6 weeks;  Exclusion of patients requiring interval slides and/or margin convergence sutures or a fixation of the subscapularis tendon | Two senior surgeons involved.  Novel single-row technique | Post-operative immobilization during 4-5 weeks in a sling. Passive and assisted active ROM started after 1 week. Strengthening exercises started after 8 weeks. |
| Nakamura et al. ^49^ | 2018 | Japan | R | 71 | Patients with all types of degenerative or traumatic tears;  Exclusion of patients with systemic disease | Number of surgeons not precisely described.  Suture bridge technique used | Duration of postoperative immobilization in a sling not precisely described. Passive ROM started after 1 week. Active assisted ROM started after 6 weeks. Strengthening exercises started after 3 months |
| Watson et al. ^69^ | 2018 | United States of America | P | 82 | Patients with supraspinatus and infraspinatus full-thickness tears;  Exclusion of patients with subscapularis repair, massive tears with only partial repair, debridement only and concomitant labral repair | Three fellowship-trained shoulder surgeons involved.  Subacromial decompression was performed in all the cases, single-row or double-row techniques used | Duration of postoperative immobilization in a sling during 4 weeks. Passive ROM started after 4 weeks. Active assisted ROM started after 4 weeks. Strengthening exercises started after 3 months |
| Basat et al. ^3^ | 2019 | Turkey | R | 176 | Patients with full-thickness rotator cuff tears having dissatisfaction after 6 months of non-operative treatment;  Exclusion of patients with instability or glenohumeral arthritis, owing to grade 3 of Patte classification, owing to grade 3 or 4 of the Goutallier classification, having diabetes, thyroid disorders or inflammatory disease, or two or more temperaments | One single surgeon involved.  Single-row or double-row according to the size of the tear. Additional procedures such as acromioplasty or biceps tenotomy performed when needed | Duration of postoperative immobilization in a sling during 4 weeks. Passive ROM started immediately after the surgery. Active assisted ROM started after 4 weeks. Strengthening exercises started between week 8 and week 12 |
| Cvetanovich et al. ^12^ | 2019 | United States of America | R | 288 | Patients with both degenerative and traumatic tears;  Exclusion of patients with superior capsular reconstruction, biologics in addition to repair or additional graft augmentation | Six surgeons involved.  Single-row or double-row repair technique | Rehabilitation protocol not precisely described |
| Haviv et al. ^27^ | 2019 | Israel | R | 97 | Patients with all types of tears with a minimal follow-up of 12 months;  Exclusion of patients with labral repair procedures, acromio-clavicular joint procedures, concurrent fractures, glenohumeral joint advanced osteoarthritis | Two surgeons involved.  Single-row repair technique | Duration of postoperative immobilization in a sling during 6 weeks. Passive ROM started immediately after the surgery. Active assisted ROM started after 6 weeks |
| Naimark et al. ^48^ | 2019 | United States of America | P | 89 | Patients with full-thickness degenerative rotator cuff tears;  Exclusion of patients with subscapularis tears | Four surgeons involved, treatment not precisely described | Rehabilitation protocol not precisely described |
| Beck et al. ^4^ | 2020 | United States of America | R | 220 | Patients with full-thickness rotator cuff tears, who failed conservative treatment;  No specific exclusion criteria | Number of surgeons involved not precisely described.  Single-row and double-row repair used | Not precisely described |
| Kim et al. ^37^ | 2020 | Korea (South) | P | 82 | Patients with all types of full and partial-thickness rotator cuff tears, etiology not described;  No specific exclusion criteria | One single surgeon involved.  Single-row or double-row repair technique used | Not precisely described |
| Sun et al. ^65^ | 2020 | China | P | 99 | Patients with small to large rotator cuff tears, with an acromiohumeral distance of more than 6 mm, a muscle fatty infiltration less than grade II and no muscle atrophy according to the tangent sign;  Exclusion of patients with arthropathy, glenohumeral joint osteoarthritis, shoulder stiffness, instability, local steroid injection within 3 months before the surgery, postoperative infection and autoimmune or systemic inflammatory diseases were excluded | One single surgeon involved, single-row or suture bridge technique using absorbable anchors | No postoperative immobilization. Passive ROM exercises started immediately after the surgery. Strengthening of the shoulder started after 3 months. |
| Tashjian et al. ^68^ | 2020 | United States of America | R | 202 | Patients with full thickness posterosuperior tears, etiology not described;  Exclusion of patients with partial-thickness repair only | Three surgeons involved.  Single-row or double-row repair techniques were used | Duration of postoperative immobilization in a sling during 6 weeks. Passive ROM started during that time. Active assisted ROM started after 6 weeks. Strengthening exercises started after 3 months |
| Gutman et al. ^25^ | 2021 | United States of America | R | 206 | Patients with full-thickness traumatic rotator cuff tears, with symptom duration less than 12 months;  Exclusion of patients with muscle atrophy of grade 2b or higher | Six surgeons involved.  Surgical technique used not precisely described | Post-operative immobilization during 6-8 weeks in a sling. Passive ROM started after 4-6 weeks. Assisted active ROM started after 4-6 weeks. Strengthening exercises started after 3 months |
| Malavolta et al. ^43^ | 2021 | Brazil | R | 275 | Patients with all types of degenerative and traumatic tears;  Exclusion of patients with isolated subscapularis tears, glenohumeral arthritis, adhesive capsulitis | Three surgeons were involved.  Single-row repair technique was used. | Post-operative immobilization during 6 weeks in a sling. Passive ROM started after 4 weeks. Strengthening exercises started after 3 months. |
| **Footnote**: R: Retrospective, ROM : Range Of Motion, P: Prospective | | | | | | | |
