# Supplementary material for: Prognostic factors for improvement of shoulder function after arthroscopic rotator cuff repair: a systematic review
Source: JSES Int. 2022 Sep 29;7(1):50–7. doi: 10.1016/j.jseint.2022.09.003 (PMC9937854; doi:10.1016/j.jseint.2022.09.003)
Supplement: Supplemental Table 2 [file mmc4.docx]

**Supplemental Table 2:** Detailed study outcomes and statistical analyses

| **Author** | **Year** | **Scale** | **Definition** | **Type** | **Time point** | **Statistical analysis method** |
| --- | --- | --- | --- | --- | --- | --- |
| Kim et al. ^36^ | 2014 | Shoulder Pain | Pain pattern, group of patients with a strictly decreasing pain pattern over all set time points, compared to the rest of the patients | D | 12 | Relationship between variables and postoperative pain pattern and pain intensity was assessed using a multiple regression analysis after a first univariable screening |
|  |  |  | Pain intensity, group of patients showing lower pain intensity than average for each follow-up period |  |  |  |
| Dwyer et al. ^20^ | 2015 | Constant-Score | Post-operative | C | 24 | Multivariable analysis using an analysis of covariance (ANCOVA) with factors chosen by the authors |
|  |  | Short WORC | Post-operative |  |  |  |
|  |  | ASES | Post-operative |  |  |  |
|  |  | Muscle strength | Post-operative strength in abduction, scapular plane (arm at 90 degrees elevation and 30 degrees in front on the coronal plane) using a dynamometer |  |  |  |
| Fermont et al. ^22^ | 2015 | Shoulder hindrance | Change | C | 12 | Multivariable analysis using linear regression with factors specifying the models chosen after univariable analysis. If P < .15, the factor was included in the final multivariable model. A check for confounding was performed |
| Pecora et al. ^54^ | 2015 | UCLA score | Post-operative | C | 12 | Multivariable linear regression with all the variables described in the paper chosen by the authors |
| Potter et al. ^56^ | 2015 | Shoulder Pain | Change | C | 12 | Multivariable analysis using multiple linear regression with factors specifying the models chosen by the authors themselves, not data-driven, no definition of factors, no explanation on how were the factors handled in the multivariable analysis |
|  |  | Simple Shoulder Test | Change |  |  |  |
|  |  | ASES | Change |  |  |  |
| Donohue et al. ^18^ | 2016 | Shoulder Pain | Change (unclear) | C | 12 | Multivariable analysis using multiple linear regression with factors specifying models chosen by authors themselves |
|  |  | ASES | Change (unclear) |  |  |  |
|  |  | Constant-Score | Change (unclear) |  |  |  |
| Tan et al. ^66^ | 2016 | Shoulder Pain | Post-operative pain frequency with activity (0 = never, 1 = monthly, 2 = weekly, 3 = daily, 4 = always) | Cat | 6 | Comparison of group traumatic vs non-traumatic, simple comparison between groups + 2-way ANOVA in order to describe the effect of trauma and time on preoperative and postoperative outcomes. |
|  |  |  | Post-operative pain severity with overhead activities (0 = none, 1 = mild, 2 = moderate, 3 = severe, 4 = very severe) |  |  |  |
|  |  | Range of Motion | Post-operative forward flexion | C |  |  |
|  |  |  | Post-operative abduction |  |  |  |
|  |  |  | Post-operative external rotation |  |  |  |
|  |  | Muscle strength | Post-operative supraspinatus strength |  |  |  |
|  |  |  | Post-operative external rotation strength |  |  |  |
|  |  |  | Post-operative internal rotation strength |  |  |  |
|  |  |  | Post-operative adduction strength |  |  |  |
|  |  | Range of Motion | Post-operative internal rotation (vertebral levels from S5 to T7) |  |  | Mann-Whitney rank sum test |
| Donohue et al. ^19^ | 2017 | Shoulder Pain | Change | C | 12 | A multiple linear regression analysis was performed with confounders chosen by the authors themselves |
|  |  | ASES | Change |  |  |  |
|  |  | Constant-Score | Change |  |  |  |
| Ohzono et al. ^51^ | 2017 | UCLA score | Satisfactory postoperative score (27) | D | 24 | Univariable analysis to evaluate the relationship between clinical parameters and the outcome. Multivariable logistic regression was performed with stepwise procedure among the clinical variables associated in univariable analysis with the outcome (p < .1) |
| Robinson et al. ^61^ | 2017 | Muscle strength | Post-operative supraspinatus strength | C | 6 | Several factors were assessed in a multiple linear regression analysis with the outcome to determine which ones were the best independent predictors. Only significant associations were reported |
|  |  |  | Post-operative external rotation strength |  |  |  |
|  |  | Range of motion | Post-operative external rotation |  |  |  |
| Chalmers et al. ^6^ | 2018 | Simple Shoulder Test | Change | C | 12 | Multivariable analysis using ordinary least square multiple linear regression with factors specifying models chosen by authors themselves |
|  |  | Shoulder Pain | Change |  |  |  |
|  |  | ASES | Change |  |  |  |
| Dierckman et al. ^17^ | 2018 | WORC | Unclear | D | 24 | Multivariable logistic regression was performed to determine the association with variables selected by the authors and supported by the literature |
| Nakamura et al. ^49^ | 2018 | Japanese Orthopedic Association | Satisfactory postoperative score (83) | D | 24 | Multivariable logistic regression analysis was performed using a stepwise technique to evaluate the significant parameters affecting the outcome. ROC curves were computed to obtain the cutoff values of the parameters affecting the outcome |
| Watson et al. ^69^ | 2018 | WORC | Change | C | 12 | Multivariable mixed linear regression model with factors chosen by the author themselves |
|  |  | ASES | Change |  |  |  |
|  |  | Shoulder Pain | Change |  |  |  |
| Basat et al. ^3^ | 2019 | Oxford Shoulder Score | Post-operative | C | 24 | Multiple linear regression with factors chosen by the authors themselves and selected after a backward elimination |
|  |  | Constant-Score | Post-operative |  |  |  |
|  |  | Shoulder Pain | Post-operative |  |  |  |
| Cvetanovich et al. ^12^ | 2019 | ASES | MCID (11.1) | D | 12 | Univariate analysis was performed with respect to each variable using Khi2 or the Student t test for categorical and continuous variables, respectively. Multivariate logistical regression analysis was performed on variables that achieved a P value of <.15 during univariable analysis. |
|  |  | Constant-Score | MCID (4.6) |  |  |  |
|  |  | SSV | MCID (29.4) |  |  |  |
|  |  | ASES | SCB (17.5) |  |  |  |
|  |  | Constant-Score | SCB (5.5) |  |  |  |
|  |  | ASES | PASS (86.7) |  |  |  |
|  |  | SSV | PASS (82.5) |  |  |  |
|  |  | Constant-Score | PASS (23.3) |  |  |  |
| Haviv et al. ^27^ | 2019 | Oxford Shoulder Score | Change | C | 24 | A multivariable stepwise regression analysis was performed to choose (reporting unclear) among the initial predictors |
| Naimark et al. ^48^ | 2019 | WORC | Change | C | 24 | Multivariate linear regression controlling for both baseline WORC score and follow-up duration. Age, sex, cuff tear index and each muscle quality factor were used as independent variables. Factors were chosen by authors themselves. |
| Beck et al. ^4^ | 2020 | ASES | Maximal outcome improvement (69.5%) | D | 12 | Stepwise multivariable logistic regression with forward selection and backward elimination procedures, with variables chosen by the authors themselves |
| Kim et al. ^37^ | 2020 | Shoulder Pain | MCID (1.5) | D | 12 | All factors associated in univariable analysis (p < 0.20) with the outcome were included in a multivariable analysis. Odds ratios of factors in the multivariable analysis were presented only if P values were inferior to 0.05 |
|  |  | ASES | MCID (21) |  |  |  |
|  |  | SSV | MCID (13) |  |  |  |
|  |  | Shoulder Pain | SCB (2.5) |  |  |  |
|  |  | ASES | SCB (26) |  |  |  |
|  |  | SSV | SCB (20) |  |  |  |
|  |  | Shoulder Pain | PASS (1.7) |  |  |  |
|  |  | ASES | PASS (78) |  |  |  |
|  |  | SSV | PASS (71) |  |  |  |
| Sun et al. ^65^ | 2020 | Range of Motion | Post-operative forward flexion | C | 24 | A multivariable linear regression model was created if at least one factor showed a significant correlation with the postoperative outcome. |
|  |  |  | Post-operative external rotation at the side |  |  |  |
| Tashjian et al. ^68^ | 2020 | Simple Shoulder Test | MCID (4.32) | D | 12 | Median regression approach was used to estimate the effects of variables chosen by the authors on outcomes |
|  |  | Shoulder Pain | MCID (2.37) |  |  |  |
|  |  | ASES | MCID (27.13) |  |  |  |
| Gutman et al. ^25^ | 2021 | ASES | Post-operative | C | 24 | Multivariable regression analysis performed on all the variables selected by the authors |
|  |  | Simple shoulder test | Post-operative |  |  |  |
|  |  | SSV | Post-operative |  |  |  |
|  |  | Shoulder Pain | Post-operative |  |  |  |
| Malavolta et al. ^43^ | 2021 | UCLA score | Change | C | 12 | Multivariable linear regression analysis with factors chosen by the authors themselves |
|  |  | ASES | Change |  |  |  |
| **Footnote**: American Shoulder and Elbow Surgeons scale: ASES, Categorized: Cat, Continuous: C, Dichotomous: D, Minimal Clinical Important Difference: MCID, Patient Acceptable Symptom State: PASS, Subjective Shoulder Value: SSV, Substantial Clinical Benefit: SCB, University of California Los Angeles scale: UCLA, Western Ontario Rotator Cuff scale: WORC | | | | | | |
