# Supplementary material for: Prognostic factors for improvement of shoulder function after arthroscopic rotator cuff repair: a systematic review
Source: JSES Int. 2022 Sep 29;7(1):50–7. doi: 10.1016/j.jseint.2022.09.003 (PMC9937854; doi:10.1016/j.jseint.2022.09.003)
Supplement: Supplemental Table 3a [file mmc6.docx]

**Supplemental Table 3a: Synthesized associations between factors and objective outcomes**

|  |  | **Significant multivariable associations** | | | |  |
| --- | --- | --- | --- | --- | --- | --- |
| **Potential prognostic factors identified** | **Number of unique analyses** | **+** | **0** | **-** | **Unclear** | **Overall quality** |
| Age (younger) | 4 ^20, 61^ | 2 | 2 |  |  | ++ |
| Concomitant rotator cuff pathologies | 1 ^20^ |  |  |  | 1 | + |
| Traumatic onset | 8 ^66^ |  | 4 | 3 | 1 | + |
| Male sex | 4 ^61, 65^ | 3 |  |  | 1 | + |
| Tear severity | 3 ^61^ |  | 3 |  |  | + |
| Tear size (smaller) | 4 ^20, 61^ | 2 | 2 |  |  | ++ |
| Difficulty with behind the back activity | 3 ^61^ |  | 3 |  |  | + |
| Difficulty with overhead activity | 3 ^61^ |  | 3 |  |  | + |
| Preoperative muscle strength | 13 ^20, 61^ | 4 | 7 | 1 | 1 | + |
| Preoperative pain level | 15 ^61^ |  | 13 | 2 |  | + |
| Preoperative range of motion | 12 ^61^ | 1 | 11 |  |  | + |
| Preoperative perceived stiffness | 3 ^61^ |  | 2 | 1 |  | + |
| Hypertension | 1 ^65^ |  | 1 |  |  | + |
| Lymphocyte monocyte ratio | 2 ^65^ | 2 |  |  |  | + |
| Operative time | 3 ^61^ |  | 3 |  |  | + |
| Preoperative overall shoulder satisfaction | 3 ^61^ |  | 3 |  |  | + |
| Repair quality | 3 ^61^ |  | 3 |  |  | + |
| Worker compensation claim | 1 ^20^ |  |  |  | 1 | + |
| Tendon mobility | 3 ^61^ |  | 3 |  |  | + |
| Tissue quality | 3 ^61^ |  | 3 |  |  | + |
| Number of anchors | 3 ^61^ | 1 | 2 |  |  | + |
| Surgical technique | 1 ^65^ |  |  |  | 1 | + |
| **Footnote**: For multivariable analyses: +, number of significant effects with a positive value (meaning the factor improves the patient’s prognosis); 0, number of non-significant effects; -, number of significant effects with a negative value (meaning factor deteriorates the patient’s prognosis).  For overall quality of evidence based on GRADE items: + very low, ++ low.  As the quality of the synthesized evidence was very limited for all the GRADE items, we focused our judgement the inconsistency items across different studies. When 50% or more of the studies had the same direction of findings, we ranked the overall quality of evidence as “low” instead of “very low”. | | | | | | |
