# Supplementary material for: Prognostic factors for improvement of shoulder function after arthroscopic rotator cuff repair: a systematic review
Source: JSES Int. 2022 Sep 29;7(1):50–7. doi: 10.1016/j.jseint.2022.09.003 (PMC9937854; doi:10.1016/j.jseint.2022.09.003)
Supplement: Supplemental Table 3b [file mmc7.docx]

**Supplemental Table 3b: Synthesized associations between factors and patient-reported outcome measures**

|  |  | **Significant multivariable associations** | | | |  |
| --- | --- | --- | --- | --- | --- | --- |
| **Potential prognostic factors identified** | **Number of unique analyses** | **+** | **0** | **-** | **Unclear** | **Overall quality** |
| Acromioclavicular joint procedures | 9 ^12, 54^ |  | 2 |  |  | + |
| Acromion type | 1 ^54^ |  | 1 |  |  | + |
| Acromioplasty | 8 ^36, 37, 43, 54^ |  | 8 |  |  | + |
| Age (older) | 42 ^6, 12, 17-20, 25, 37, 43, 48, 51, 54, 56, 68, 69^ | 3 | 34 | 1 |  | + |
| Alcohol use | 1 ^17^ |  | 1 |  |  | + |
| American Society of Anesthesiologists classification | 6 ^6, 56^ |  | 5 |  | 1 | + |
| Biceps procedures | 18 ^4, 6, 12, 37, 43, 51, 54^ |  | 10 |  | 4 | + |
| Body mass index (increasing) | 22 ^6, 12, 22, 25, 56, 69^ | 1 | 15 | 1 |  | + |
| Charlson Comorbidity Index | 2 ^37^ |  | 2 |  |  | + |
| Complete repair | 2 ^43^ |  | 2 |  |  | + |
| Concomitant rotator cuff pathologies | 3 ^20^ |  |  |  | 3 | + |
| Concomitant procedures | 3 ^19^ |  | 3 |  |  | + |
| Cuff tear index | 1 ^48^ |  | 1 |  |  | + |
| Depression and anxiety | 6 ^56^ |  | 5 | 1 |  | + |
| Diabetes | 15 ^12, 17, 43, 51, 69^ |  | 5 | 2 |  | + |
| Dominance affected side | 12 ^4, 12, 43, 51^ |  | 4 | 1 |  | + |
| Fatty infiltration | 12 ^6, 22, 48, 51^ |  | 7 | 3 |  | + |
| Follow-up duration | 1 ^51^ |  |  |  |  | + |
| Hypertension | 11 ^12, 43, 51^ |  | 3 |  |  | + |
| Infraspinatus repair | 2 ^43^ |  | 2 |  |  | + |
| Lateral debridement | 8 ^12^ |  | 1 |  |  | + |
| Male sex | 35 ^12, 19, 25, 37, 43, 48, 54, 56, 68, 69^ | 1 | 22 | 2 | 1 | + |
| Mobilization | 8 ^12^ |  | 1 |  |  | + |
| Number of anchors | 4 ^6, 17^ |  | 4 |  |  | + |
| Postoperative retear | 2 ^37^ |  | 1 | 1 |  | + |
| Posterosuperior tear repair | 2 ^54^ |  | 2 |  |  | + |
| Postoperative shoulder stiffness | 1 ^36^ |  |  | 1 |  | + |
| Preoperative corticosteroid injections | 3 ^18^ | 3 |  |  |  | + |
| Preoperative functional scores (lower) | 29 ^12, 20, 25, 27, 37, 43, 51^ | 17 | 4 |  | 3 | ++ |
| Preoperative muscle strength | 4 ^51^ |  |  |  |  | + |
| Preoperative pain level (higher) | 7 ^36, 37, 49, 51^ | 4 |  |  |  | ++ |
| Preoperative physical therapy | 6 ^18, 19^ |  |  |  | 6 | + |
| Preoperative range of motion (higher) | 2 ^22, 49^ | 1 |  |  |  | + |
| Procedure location | 3 ^6^ |  |  |  | 3 | + |
| Repair technique | 12 ^6, 12, 37^ |  | 6 |  | 2 | + |
| Smoking status | 24 ^6, 12, 17, 37, 43, 51, 56, 69^ | 1 | 14 | 1 |  | + |
| Subscapularis repair | 5 ^6, 43^ |  |  | 2 | 3 | + |
| Supraspinatus repair | 2 ^43^ |  |  |  | 2 | + |
| Surgeon effect | 1 ^17^ |  |  |  | 1 | + |
| Symptom duration (shorter) | 13 ^18, 19, 25, 36, 51^ | 6 | 6 |  |  | ++ |
| Synovitis | 2 ^36^ |  | 2 |  |  | + |
| Traumatic onset | 9 ^18, 19, 51, 66^ |  | 4 | 2 |  | + |
| Tear location | 6 ^18, 19^ |  | 6 |  |  | + |
| Tear pattern | 12 ^4, 12, 36, 37^ |  | 4 |  |  | + |
| Tear retraction | 10 ^6, 22, 43, 51, 56^ |  | 9 | 1 |  | + |
| Tear shape | 3 ^69^ |  | 3 |  |  | + |
| Tear size | 45 ^6, 12, 17, 18, 20, 25, 27, 36, 37, 51, 54, 56, 68, 69^ |  | 39 | 3 | 3 | + |
| Temperament | 3 ^3^ |  | 3 |  |  | + |
| Timing of preoperative corticosteroid injection | 3 ^19^ |  |  |  | 3 | + |
| Worker compensation claim (absence) | 20 ^4, 12, 20, 25, 51, 68^ | 9 | 5 |  |  | ++ |
| **Footnote**: For multivariable analyses: +, number of significant effects with a positive value (meaning the factor improves the patient’s prognosis); 0, number of non-significant effects; -, number of significant effects with a negative value (meaning factor deteriorates the patient’s prognosis).  For overall quality of evidence based on GRADE items: + very low, ++ low.  As the quality of the synthesized evidence was very limited for all the GRADE items, we focused our judgement the inconsistency items across different studies. When 50% or more of the studies had the same direction of findings, we ranked the overall quality of evidence as “low” instead of “very low”. | | | | | | |
