# Supplementary material for: Prognostic factors for improvement of shoulder function after arthroscopic rotator cuff repair: a systematic review
Source: JSES Int. 2022 Sep 29;7(1):50–7. doi: 10.1016/j.jseint.2022.09.003 (PMC9937854; doi:10.1016/j.jseint.2022.09.003)
Supplement: Supplemental Table 4 [file mmc8.docx]

**Supplemental Table 4: Risk of bias of included studies**

| **Author** | **Year** | **Study participation** | **Study attrition** | **Prognostic Factor measurement** | **Outcome measurement** | **Study confounding** | **Statistical analysis and reporting** | **Overall** |
| --- | --- | --- | --- | --- | --- | --- | --- | --- |
| Kim et al. ^36^ | 2014 | Moderate | Moderate | High | High | Moderate | High | **High** |
| Dwyer et al. ^20^ | 2015 | Moderate | High | High | Low | Moderate | High | **High** |
| Fermont et al. ^22^ | 2015 | Moderate | Low | Moderate | Low | High | High | **High** |
| Pecora et al. ^54^ | 2015 | Moderate | High | High | Moderate | Moderate | High | **High** |
| Potter et al. ^56^ | 2015 | High | Moderate | Moderate | High | Moderate | High | **High** |
| Donohue et al. ^18^ | 2016 | Low | Moderate | Moderate | High | Moderate | High | **High** |
| Tan et al. ^66^ | 2016 | Moderate | Moderate | Moderate | High | High | High | **High** |
| Donohue et al. ^19^ | 2017 | High | High | High | Moderate | High | High | **High** |
| Ohzono et al. ^51^ | 2017 | Moderate | Moderate | Moderate | High | Moderate | High | **High** |
| Robinson et al. ^61^ | 2017 | High | High | Moderate | Moderate | High | High | **High** |
| Chalmers et al. ^6^ | 2018 | Moderate | High | Moderate | Low | Moderate | Moderate | **High** |
| Dierckman et al. ^17^ | 2018 | High | High | High | High | Moderate | High | **High** |
| Nakamura et al. ^49^ | 2018 | Low | High | Moderate | Moderate | Low | High | **High** |
| Watson et al. ^69^ | 2018 | Low | Moderate | Moderate | Moderate | Moderate | Low | **Moderate** |
| Basat et al. ^3^ | 2019 | High | High | High | Moderate | High | High | **High** |
| Cvetanovich et al. ^12^ | 2019 | High | Moderate | High | High | Moderate | Moderate | **High** |
| Haviv et al. ^27^ | 2019 | Moderate | High | High | Low | Moderate | High | **High** |
| Naimark et al. ^48^ | 2019 | Moderate | Moderate | Moderate | High | Moderate | Moderate | **High** |
| Beck et al. ^4^ | 2020 | Moderate | High | Moderate | Low | Low | Moderate | **High** |
| Kim et al. ^37^ | 2020 | Moderate | Moderate | Low | Low | Moderate | Moderate | **Moderate** |
| Sun et al. ^65^ | 2020 | Moderate | Moderate | High | Low | High | High | **High** |
| Tashjian et al. ^68^ | 2020 | Moderate | High | Low | Moderate | Low | High | **High** |
| Gutman et al. ^25^ | 2021 | Moderate | Low | Moderate | Low | Moderate | Moderate | **Moderate** |
| Malavolta et al. ^43^ | 2021 | High | Moderate | High | Moderate | Moderate | High | **High** |
