# Supplementary material for: Prognostic factors for improvement of shoulder function after arthroscopic rotator cuff repair: a systematic review
Source: JSES Int. 2022 Sep 29;7(1):50–7. doi: 10.1016/j.jseint.2022.09.003 (PMC9937854; doi:10.1016/j.jseint.2022.09.003)
Supplement: Supplemental Table 5 [file mmc9.docx]

**Supplemental Table 5: Detailed study modelling phase**

| **Author** | **Year** | **Factors selection process** | **Initial set of factors** | **Decision to include in a model** | **Outcome** | **Final set of factors** | **Model performance** |
| --- | --- | --- | --- | --- | --- | --- | --- |
| Chalmers et al. ^6^ | 2018 | Factors chosen by authors | Age, Body Mass Index (BMI), Smoking, American Society of Anesthesiologists classification, Repair technique, Subscapularis tear repair, Fatty infiltration of supraspinatus muscle, Fatty infiltration of infraspinatus muscle, Procedure location, Tear size, Number of anchors, Tear retraction, Biceps procedures | Factors presented only if significant in a multivariable analysis | Change in Simple Shoulder Test (SST) | **Biceps procedures*** | - |
|  |  |  |  |  | Change in shoulder pain | **Age*, Smoking status*, Biceps procedures*** | - |
|  |  |  |  |  | Change in American Shoulder Elbow Scale (ASES) | **BMI*, Fatty infiltration of infraspinatus muscle*, Tear size*, Biceps procedures*** | - |
| Watson et al. ^69^ | 2018 | Factors chosen by authors | Age, Sex, BMI, Smoking, Diabetes, Tear shape, Tear size | All initial factors were included in the models | Change in ASES | Age, **Sex***, BMI, Smoking, **Diabetes***, Tear shape, Tear size | - |
|  |  |  |  |  | Change in shoulder pain | Age, **Sex***, BMI, Smoking, Diabetes, Tear shape, Tear size | - |
|  |  |  |  |  | Change in Western Ontario Rotator Cuff (WORC) | Age, **Sex***, BMI, Smoking, **Diabetes***, Tear shape, Tear size | - |
| Cvetanovich et al. ^12^ | 2019 | Factors chosen by authors | Age, Sex, BMI, Smoking, Tear size, Tear pattern, Surgical technique, Biceps procedures, Acromioclavicular joint procedures, Affected side dominant,  Mobilization, Worker compensation claim, Diabetes, Hypertension, Lateral debridement, Preoperative functional scores | Factors were included if p value was inferior than < .15 on univariable analysis | ASES Minimal Clinically Important Difference (MCID) | Age, **Preoperative ASES*, Smoking status***, Biceps procedure, Single-row repair | - |
|  |  |  |  |  | Constant MCID | Age, **Preoperative Constant***, Biceps procedure, Single-row repair | - |
|  |  |  |  |  | Simple Shoulder Value (SSV) MCID | **Preoperative SSV***, Mobilization, Massive tear, Distal clavicle excision, Dominant side | - |
|  |  |  |  |  | ASES Substantial Clinical Benefit (SCB) | Age, **Preoperative ASES***, **Worker compensation claim ***, Biceps procedure, Single-row repair | - |
|  |  |  |  |  | Constant SCB | Age, **Preoperative Constant***, Diabetes, Biceps procedure, **Single-row repair*** | - |
|  |  |  |  |  | ASES PASS | Age, BMI, Preoperative ASES, **Worker compensation claim***, Comorbid hypertension, Dominant side | - |
|  |  |  |  |  | SSV PASS | **Worker compensation claim***, Labral debridement, BMI | - |
|  |  |  |  |  | Constant PASS | **Preoperative Constant***, **Worker compensation claim***, Biceps procedure, Distal clavicle excision, Massive tear, Age, **BMI*** |  |
| Naimark et al. ^48^ | 2019 | Factors chosen by authors | Controlling for: Baseline WORC score, Follow-up duration  Predictors: Age, Sex, Cuff tear index, Each muscle quality, Muscle atrophy, Fatty infiltration | All initial factors | Change in WORC | Age, Sex, Cuff Tear Index, **Tangent sign***, Supraspinatus atrophy, Supraspinatus fatty infiltration, Infraspinatus atrophy, Infraspinatus fatty infiltration | - |
| Beck et al. ^4^ | 2020 | Factors chosen by authors | Comorbidities, Smoking status, Age, Sex, BMI, Dominant side, Symptom duration, Biceps tenodesis, Distal clavicle excision, Glenohumeral debridement, Tear size | Stepwise regression with forward and backward selection on all initial variables | Maximal outcome improvement in ASES | Biceps tenodesis, **Dominant side***, Infraspinatus involvement, **Worker compensation claim*** | - |
| Kim et al. ^36^ | 2020 | Factors chosen by authors | Age, Sex, BMI, Bone Mineral Density, Dominant side, Smoking status, Charlson Comorbidity Index, Tear thickness, Tear size, Fatty infiltration, Subscapularis muscle involvement, Subscapularis tear repair, Repair technique, Acromioplasty, Biceps procedure, Retear, Baseline functional score | Factors were included if p value for univariable association was inferior than 0.20. | Visual Analog Scale (VAS) pain MCID | Sex, Tear size, Subscapularis tear repair, **Baseline VAS pain scale*** | Hosmer-Lemeshow test  p = 0.623 |
|  |  |  |  |  | ASES MCID | Sex, Smoking status, Tear size, Acromioplasty, **Retear***, **Baseline ASES score*** | Hosmer-Lemeshow test  p = 0.529 |
|  |  |  |  |  | SSV MCID | Smoking status, Tear size, Acromioplasty, Charlson Comorbidity Index, **Baseline SSV score*** | Hosmer-Lemeshow test  P = 0.255 |
|  |  |  |  |  | University California Los Angeles (UCLA) score MCID | Age, **Tear size***, Retear, Charlson Comorbidity Index, **Baseline UCLA score*** | Hosmer-Lemeshow test  P = 0.704 |
|  |  |  |  |  | VAS pain SCB | Sex, Tear size, Subscapularis tear repair, Biceps procedure, BMI, **Baseline VAS pain scale*** | Hosmer-Lemeshow test  P = 0.667 |
|  |  |  |  |  | ASES score SCB | Sex, Tear size, Acromioplasty, **Baseline ASES score*** | Hosmer-Lemeshow test  P = 0.300 |
|  |  |  |  |  | SSV score SCB | Tear size, Acromioplasty, Charlson Comorbidity Index, **Baseline SSV score*** | Hosmer-Lemeshow test  P = 0.336 |
|  |  |  |  |  | UCLA score SCB | **Age***, Charlson Comorbidity Index, Tear thickness, Tear size, Acromioplasty, Biceps procedure, **Baseline UCLA score*** | Hosmer-Lemeshow test  P = 0.329 |
|  |  |  |  |  | VAS pain Patient Acceptable Symptom State (PASS) | **Sex***, Age, Smoking status, **Biceps procedure***, Retear, **Baseline VAS pain scale*** | Hosmer-Lemeshow test  P = 0.592 |
|  |  |  |  |  | ASES score PASS | Sex, **Age***, **Baseline ASES score*** | Hosmer-Lemeshow test  P = 0.340 |
|  |  |  |  |  | SSV score PASS | Repair technique, **Baseline SSV score*** | Hosmer-Lemeshow test  P = 0.155 |
|  |  |  |  |  | UCLA score PASS | Sex, **Age***, Charlson Comorbidity Index, **Baseline UCLA score*** | Hosmer-Lemeshow test  P = 0.538 |
| Gutman et al. ^25^ | 2021 | Factors chosen by authors | Age, BMI, Sex, Tear area, Baseline functional score, Worker compensation claim, Time to surgery | All initial factors | Postoperative ASES | Age, BMI, Sex, Tear area, Baseline functional score, **Worker compensation claim***, **Time to surgery*** | - |
|  |  |  |  |  | Postoperative SST | Age, BMI, **Sex***, Tear area, Baseline functional score, Worker compensation claim, **Time to surgery*** | - |
|  |  |  |  |  | Postoperative SSV | Age, BMI, Sex, Tear area, Baseline functional score, Worker compensation claim, **Time to surgery*** | - |
|  |  |  |  |  | Postoperative VAS pain | Age, BMI, Sex, Tear area, **Baseline functional score***, Worker compensation claim, **Time to surgery*** | - |
| **Footnote**: * factor significant in multivariable analysis  American Shoulder and Elbow Surgeons scale: ASES, Minimal Clinical Important Difference: MCID, Patient Acceptable Symptom State: PASS, Subjective Shoulder Value: SSV, Substantial Clinical Benefit: SCB, University of California Los Angeles scale: UCLA, Visual Analog Scale: VAS, Western Ontario Rotator Cuff scale: WORC | | | | | | | |
